# Bayesian Assessment of the Accuracy of a PCR-Based Rapid Diagnostic Test for Bovine Tuberculosis in Swine

**DOI:** 10.3389/fvets.2019.00204

**Published:** 2019-06-26

**Authors:** Soledad Barandiaran, María Sol Pérez Aguirreburualde, María Jimena Marfil, Marcela Martínez Vivot, Natalia Aznar, Martín Zumárraga, Andrés M. Perez

**Affiliations:** ^1^Universidad de Buenos Aires, Facultad de Ciencias Veterinarias, Cátedra de Enfermedades Infecciosas, Buenos Aires, Argentina; ^2^Consejo Nacional de Investigaciones Científicas y Técnicas, Buenos Aires, Argentina; ^3^Veterinary Population Medicine Department, Center for Animal Health and Food Safety, College of Veterinary Medicine, University of Minnesota, St. Paul, MN, United States; ^4^Instituto de Patobiología, Veterinaria, UEDD INTA-CONICET, Buenos Aires, Argentina; ^5^Instituto de Agrobiotecnología y Biología Molecular, UEDD INTA-CONICET, Buenos Aires, Argentina

**Keywords:** tuberculosis, swine, diagnosis, bacteriology culture, PCR, bayesian modeling

## Abstract

Infection with the *Mycobacterium bovis (M. bovis)* causes a disease referred to as bovine tuberculosis (bTB), which affects a wide range of mammal hosts. Many countries have implemented control and eradication plans that have resulted in variable levels of efficacy and success. Although bTB is a notifiable disease in Argentina, and a control plan that targets cattle herds has been in place for decades, *M. bovis* is still prevalent in cattle, swine, and certain wild species. The aim of the paper here was to assess the sensitivity (Se), specificity (Sp), and positive and negative predictive values (PPV and NPV) of PCR from tissue, which is a test for rapid *M. bovis* detection in swine. Bacteriological culture was also performed for comparison purposes. A Bayesian approach was applied to estimate the accuracy of the diagnostic tests, PCR and bacteriological culture, in 266 swine samples with bTB-like lesions recovered during routine official inspections at slaughterhouses. A one-population model, assuming conditional dependence between test results, and incorporating prior information on the performance of the tests obtained from the literature, was used to estimate the tests Se and Sp. The accuracy of the combined (in parallel) application of both tests was also estimated. The Se of the PCR (82.9%) was higher than the Se of the bacteriological culture (79.9%), whereas the Sp of both tests was similar (88.5 and 89.0%, respectively). Furthermore, when both techniques were assessed in parallel, the Se of the diagnostic system increased substantially (Se = 96.6%) with a moderate Sp loss (Sp = 78.8%; PPV = 92.8%; NPV = 89%). Results suggest that the PCR, or the combined application of bacteriological culture and PCR, may serve as an accurate diagnostic tool to confirm bTB in swine samples. Results here will help the design and implementation of effective surveillance strategies for the disease in swine of Argentina and other settings in which the disease is prevalent.

## Introduction

*Mycobacterium bovis* (*M. bovis*) is a member of the *Mycobacterium tuberculosis* complex (MTC) (1). MTC members cause tuberculosis (TB) in a wide range of host species worldwide, and *M. bovis* is a major causative agent of bovine tuberculosis (bTB), holding a significant zoonotic potential (2). Globally, bTB prevalence is quite heterogeneous and somehow related to the social features of the setting, with the disease being endemic in most developing countries and eradicated from many developed regions (3). Factors suggested to have impaired the efficacy of bTB control programs include limited political willingness, resources scarcity, existence of wildlife reservoirs, and limitations of the available diagnostic tests (4–8).

There are many tests available and widely used to diagnose the disease (9, 10). Many bTB diagnostic tests have been applied for decades, and rely on a variety of biological principles, including the measurement of the cellular response in the host following the application of an intradermal test, the histopathology of postmortem specimens, or the bacteriological culture (BC) of the agent (11). Currently, there are many other testing strategies available, such as the interferon gamma essay, antibody-based assays, or the detection of bTB DNA by PCR, which presents new opportunities to improve or develop control plans, but for which there is still a need to gain understanding of their performance in field conditions (9, 12, 13). However, because those diagnostic techniques have values of sensitivity (Se) and specificity (Sp) that vary on each animal species and the specific epidemiological situation, estimation of the test accuracy is challenging (14–16).

In Argentina, bTB is endemic in both livestock and wildlife populations (17). The protein purified derivative (PPD) skin test and the meat inspection of carcasses at slaughterhouses are the actions approved and used in the bTB's Control and Eradication National plan (SENASA, Res.128/2012). However, PPD testing is compulsory only for dairy cattle, dairy goats, dairy sheep, and genetic nuclei and multipliers. Control activities are voluntary for other species, including swine.

Official records estimated that 0.3% of inspected pigs in Argentina showed TB-like lesions, as observed by the Argentine Animal Health Service (SENASA) inspectors at slaughterhouses. However, evidence suggests that the figure may have been underestimated (18–20).

The BC is considered the reference technique for bTB diagnosis, even though the Se of the test is only ~80% (10, 12), impairing its systematic application on disease control programs. Moreover, it is a laborious technique, which requires high biosecurity facilities and relatively specialized workforce for implementation. Also, because the technique depends on the agent's viability, preservation, and quality of the collected sample drastically affects the results (10, 21). Another key limitation is the relatively long turnaround time of the techniques (on average, between 2 and 3 months), which jeopardizes the ability to inform decision-makers on a timely manner (22, 23). For that reason, the BC has important limitations as a confirmatory test for the macroscopic inspection at slaughterhouses (Table 1).

**Table 1:** Key features of both diagnostic tests that influence the feasibility of implementation in the context of a control plan.

**Test**	**Bacteriological culture + identification by PCR**	**DNA extraction from tissue + identification by PCR**
**Requirements**		
Laboratory biosafety	High: BSL-2. During all process. Special features: Viability and exponential amplification of the agent. Long process. High exposition risks.	High: BSL-2 For initial manipulation. Until extraction and inactivation of the remaining material.
Average turnaround time	2–3 months	3–4 days
Relative cost	1*	1.33^#^
Trained personnel	Highly dependent on the operator skills (technique sensitivity varies with the level of skills and experience of the operator)	Average training requirements. Allows broader automation. PCR tests are more robust and versatile tests for labs settings.

*Relative cost: based on the comparison between average costs provided by 3 local labs*.

* *cost of Mycobacteriology culture includes: culture, Ziehl-Neelsen staining, and identification by PCR*.

# *cost of direct PCR from tissue samples, includes: extraction kit and PCR*.

The direct PCR analysis from tissue samples has been developed as an alternative technique to obtain a relatively fast confirmation of the infection. Direct PCR is believed to allow for the rapid, specific detection of *Mycobacteria*, and it is independent of the agent's viability on the sample. Some studies have reported the performance of the direct PCR in samples from cattle, buffaloes, humans, and some wildlife species (10, 14, 16). However, to the best of the authors' knowledge, the accuracy of the test is yet-to-be-assessed in swine (12).

The evaluation of diagnostic test performance, traditionally, has been based on the comparison of test results against a gold standard, allowing the assessment and validation of new techniques in comparison to a reference test. The limitation of such analytical approach is that the assumption of perfect Se and Sp of the reference test is, typically, questionable for many diseases, including bTB (21). Alternatively, Bayesian methods have been proposed as an analytical option to assess the accuracy of diagnostic tests without the requirement of a reference test (24). Bayesian methods have previously been used to estimate the accuracy of TB diagnostic techniques in bovine populations (8, 10, 25).

The aim of the study here was to estimate the Se and Sp of the BC, and of a rapid diagnostic test (PCR from tissue) on swine TB-like lesions obtained at slaughterhouses, and thereafter to evaluate the combined performance of those tests. Results will inform current discussions regarding the evaluation and potential modifications to the bTB control strategies in the target population and in the context of the Argentine disease control plan. Results may also be useful for countries in which bTB is prevalent in swine populations.

## Materials and Methods

### Sample Collection

Swine samples (*n* = 266) showing bTB-like lesions (TBL) were collected in multiple visits to three slaughterhouses located in the Province of Buenos Aires between 2015 and 2017. Those three slaughterhouses processed pigs from the main productive region of Argentina, which includes the provinces of Buenos Aires, Córdoba, Santa Fe, La Pampa, and Entre Ríos. Approximate 4 × 4 cm cuts of lymph nodes showing bTB-like lesions were collected. Additionally, tissue samples from swine shipped from bTB-free premises were also collected in order to validate the DNA extraction and PCR assay.

All samples were stored at −20°C. BC and PCR were carried out at the Infectious Disease Department's Mycobacterial diagnosis laboratory of the Veterinary School of the University of Buenos Aires. Because samples were collected from animals inspected post-mortem by the national authority and according to national regulations, no ethical or farmer's consent approval was required.

### Diagnostic Procedures

#### Preparation of the Samples for PCR and Culture

Samples (4–7 g) of each individual lymph node were placed into a mortar and crushed with sterile sand and 10 mL of sterile bi-distilled water for homogenization. Two milliliters of this homogenate were transferred into a 15 mL tube and 4 mL of 4% NaOH were added to decontaminate the sample using the Petroff's modified method described elsewhere (26). A portion (~400 μL) of the homogenate was separated and frozen at −20°C for further DNA extraction.

#### DNA Extraction

Invitrogen™ PureLink™ Genomic DNA Mini Kit (Invitrogen, California, USA) was used for DNA extraction directly from tissue, according to the manufacturer's protocol. The obtained DNA was stored at −20°C until use for the PCR assay.

#### Bacteriological Culture

Bacteriological culture was performed following a protocol established elsewhere (26). Stonebrink and Löwenstein Jensen media were inoculated and incubated up to 60 days at 37°C and examined every 2 weeks. When bacterial growth was observed, Ziehl Neelsen staining was performed to observe acid fast bacilli, and, if positive staining, a loop full of bacteria was suspended in 200 μL of bi-distilled water and thermal lysis was performed at 95°C for 45 min. Lysates obtained were stored at −20°C until PCR assay.

#### Polymerase Chain Reaction (PCR) Assay for *M. Bovis* Detection

PCR was conducted in both DNA extracted from tissue and colonies lysates, for the detection of the insertion sequence *6110* (IS*6110*) characteristic of the MTC (27). Positive (bTB-confirmed sample) and negative controls (bi-distilled water) were also evaluated by the PCR. The primers used for the amplification, as well as PCR cycling conditions, have been described elsewhere (28). Colonies grown in Stonebrink media and IS*6110*-PCR positive were subject to spoligotyping to identify and to type the *M. bovis* isolates, following the protocol described by Kamerbeek et al. (29). Spoligotyping was carried out using the spoligotyping kit (Mapmygenome India). *M. tuberculosis* H37Rv (ATCC 27294) and *M. bovis* Bacillus Calmette-Guerin (BCG) (ATCC 27289) were included as reference strains for each assay.

#### Detection of *Mycobacteria* other than Tuberculosis

Ziehl Neelsen staining-positive isolates that were IS*6110-*PCR negative, were tested for its identification. The IS*1245*-PCR was used to detect the *Mycobacterium avium (M. avium)* complex (30). PCR controls were also conducted using a strain of *M. avium* obtained from a pure culture by thermal lysis as a positive control and bi-distilled water as a negative control.

### Statistical Model

#### Latent Class Analysis

A Bayesian approach was used to estimate the Se and Sp of the BC and the PCR test (24) in samples showing bTB-like lesions (*n* = 266) and in the absence of a gold standard. Samples were considered to have originated from one single population, given that only samples showing bTB-like lesions were evaluated. For the analysis, results from both tests were assumed to be conditionally dependent because, although biological principles of both tests are different (the culture required that the pathogenic agent was viable, whereas the PCR only requires the presence of the genetic material in sufficient quantity), both tests are based on the detection of the mycobacteria. For that reason, we preferred to follow the conservative assumption that results were not independent.

Prior distributions for model parameters (including test Se and Sp and the disease prevalence, p) were initially approximated using information on the expected values and uncertainty around that expectation, from data reported in the peer-reviewed literature (Table 2). Beta distributions of the parameters were fitted using BetaBuster (https://betabuster.software.informer.com/), using a most likely value based on the median of estimates published in the literature, and a lower bound of the credibility interval that was approximately three standard deviations below the median, according to the published data (lower 99%CI Se = 0.58; Sp = 0.81). We preferred to use wide prior standard deviations to reflect the uncertainty around the true value of the parameters, considering that uncertainty related to the true value of the parameters is likely larger than simply the 95% CI of the results reported in the literature. Uniform distributions were used for the two co-variances (33). Field data were then used to modify the prior distributions and estimate posterior distributions in a Bayesian framework, using a one-population model and assuming conditional dependence between test results. Posterior distributions were reported as the posterior estimates of the median and posterior probability intervals (95% PPI). The code is provided as Supplementary Table 2. All analyses were implemented in the WinBUGS software, version 1.4, and results were computed for 10,000 iterations, after the first 1,000 were burnt-in. Autocorrelation was eliminated through thinning the chains by collecting one in 10 consecutive samples (https://www.mrc-bsu.cam.ac.uk/software/bugs/the-bugs-project-winbugs/). The influence of the selected priors on the posterior distributions was evaluated by comparing the initial models with a model fitted using non-informative uniform (0.1) distributions alternatively for the parameters of each test and the prevalence (Supplementary Table 1). The outputs including the MCMC trace-plots, posterior density distribution plots and convergence were visually assessed (Supplementary Figure 1).

**Table 2:** Parameters of the beta distribution and source of data used to estimate the accuracy of both bTB tests in swine samples from Argentina.

**Parameter**		**Most likely value**	**Uncertainty**	**References**	**Parameters of the beta distribution**	
					**A**	**B**
Bacteriology culture	SeBC	0.79	>0.6	(10, 31)	16.1034	5.0197
	SpBC	0.97	>0.8	(10, 31)	17.2976	1.5041
PCR	SePCR	0.81	>0.6	(10, 12, 14, 15)	13.7759	3.7573
	SpPCR	0.99	>0.8	(10, 12)	14.52192	1.13658
True prevalence	P	0.32	>0.2	(32)	5.025	7.0375

*BC and MTC-PCR IS6110 (PCR). Uncertainty was measured as the 95% confidence level that the parameter was higher (>) than a certain value. Se, Sensitivity; Sp, Specificity*.

Agreement between the results obtained from both test was measured using the kappa statistic. The Kappa coefficient, combined Se and Sp of the tests used in series and in parallel, and the positive and negative predictive values of the tests were calculated using the posterior estimates of the model and using the WinEpi software (34) as:

Se series = Se_PCR_ × Se_BC_

Se parallel = 1–(1–Se_PCR_) × (1–Se_BC_)

Sp(series) = 1– (1–Sp_PCR_) × (1–Sp_BC_)

Sp(parallel) = Sp_PCR_ × Sp_BC_

For presenting the results here, we followed the guidelines for reporting of diagnostic accuracy in studies that use Bayesian Latent class models (STARD-BLCM) described elsewhere (35).

## Results

### Descriptive Results

Most (171/266, 64.8%) samples were culture-positive, and most of those samples (137/171, 80.1%) were also MTC-IS*6110*+ PCR-positive. Out of the PCR-positive samples (176/266, 66.2%), only some (39/176, 15%) were BC-negative. A few (13/56, 23.2%) of the remaining 21% culture and PCR-negative samples (i.e., 4.9% of all the samples) were *M. avium complex* (IS*1245*+)-positive (Table 3). All IS*6110*-positive samples showed spoligotypes that were characteristic of *M. bovis* species, due to the absence of the 3, 9, 16, and 39–43 spacers.

**Table 3:** Distribution of the results for both bTB diagnostic tests applied.

	**TB positive samples**			**TB negative samples**	
	**POS_**BC**_/POS_**PCR**_**	**POS_**BC**_/NEG_**PCR**_**	**NEG_**BC**_/POS_**PCR**_**	**NEG_**BC**_/NEG_**PCR**_**	**Total**
TBL	137	34	39	56	266
	51%	13.8%	15%	21%	100

*Samples of swine lymph nodes with TBL obtained from slaughterhouses were processed by BC and MTC-PCR IS6110 +PCR*.

### Estimation of Tests Se and Sp

The estimated (posterior) Se of the bacteriological culture and of the PCR were 79.9% (95% posterior probability intervals, PPI: 71.69–88.7%) and 82.9% (95% PPI: 74.35–92.3%), respectively. The estimated (posterior) Sp was similar for both tests, with a value of 88.5% (95% PPI: 67.2–99.5%) for culture, and of 89.05% (95% PPI: 69.8–99.1%) for PCR.

Bovine tuberculosis prevalence in TB-like samples was 74.39% (95% PPI: 63.3–83.5%). The agreement of both tests was moderate (Kappa coefficient = 0.395; 95% CI (confidence interval) = 0.304–0.486). The negative and positive posterior correlation estimated between the diagnostic tests was uncertain, −0.02 (95% PPI: −0.2–0.33) and 0.16 (95% PPI: −0.15–0.74), respectively. The low correlations between the two test Se and between the two test Sp for samples showing bTB-like lesions suggests that the results of both tests were independent from each other.

**Figure 1:**
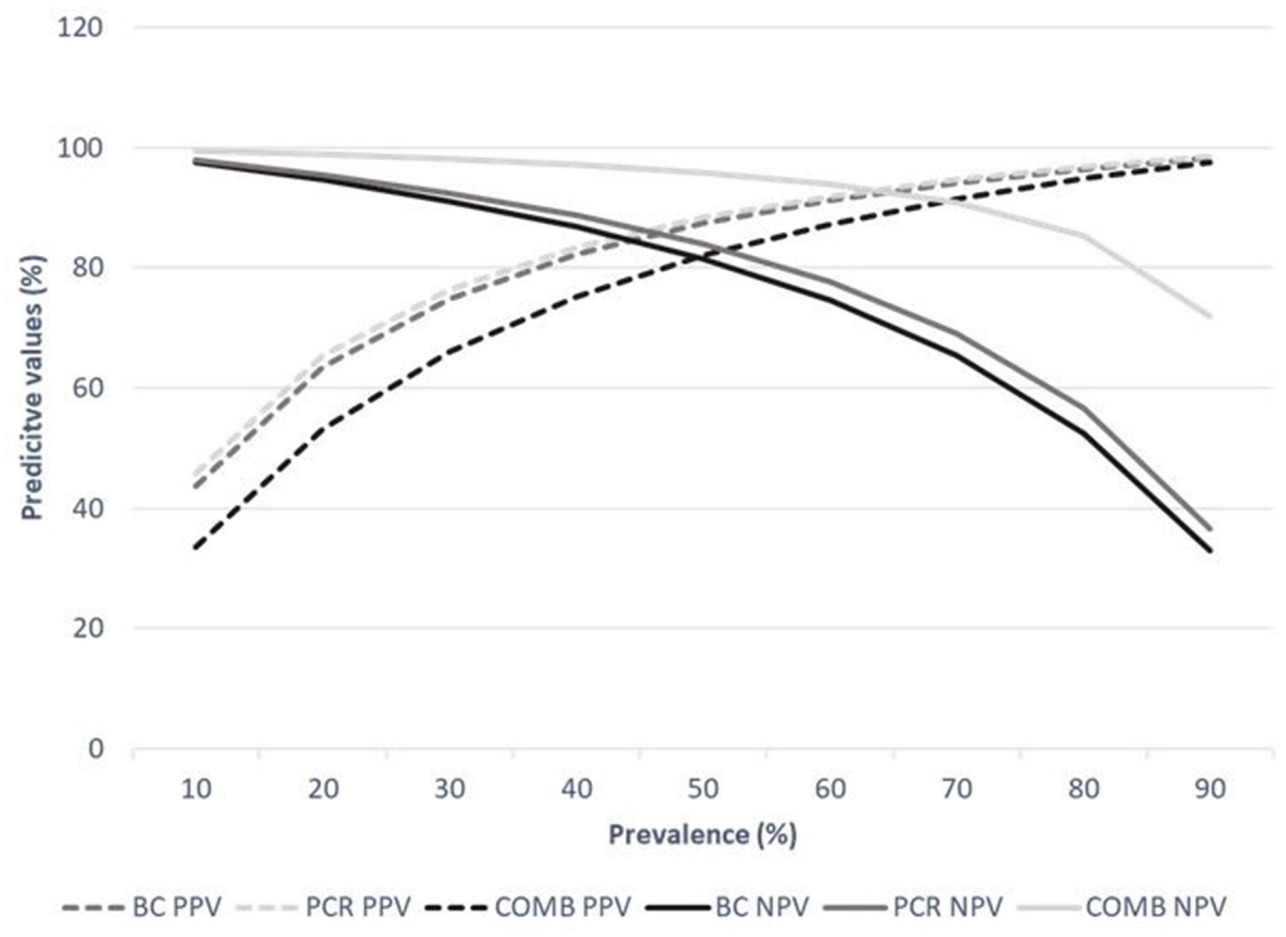
Estimates of predictive values for a range of prevalence values based on the Se and Sp obtained in the analyses presented here. PPV, Positive predictive value; NPV, Negative predictive value; BC, Bacteriological culture; PCR, Polymerase Chain Reaction; COMB, Combined techniques in parallel.

Combination of both tests was evaluated considering the estimated prevalence, obtaining a Se and Sp of 66.2 and 98.7% (positive predictive value, PPV = 92.8%; negative predictive value, NPV = 89%) when the test were combined in series, and a Se and Sp of 96.6 and 78.8% (PPV = 92.8%; NPV = 89%) when combined in parallel. Results for a broad range of hypothetical prevalence values are presented in Figure 1 to illustrate the expected variation on the PPV and NPV for alternative scenarios of disease prevalence when those two techniques were combined in parallel.

Results were not sensitive to the selection of the prior distributions, as suggested by the relatively consistency (magnitudes of percent differences <9%) in the results when using non-informative priors (Supplementary Table 1), to the posterior distribution of Se and Sp for both tests except for the Sp of the tests in which a reduction of 26.6% for the culture and 29.1 for the PCR was estimated.

## Discussion

Although significant improvements have been made in the last 20 years, control and eradication of bTB continues to be a major challenge for Latin American countries (36, 37). Furthermore, the disease has re-emerged in humans in the region, reinforcing the need to understand the role of domestic and wild animals on the disease epidemiology, and, most importantly, the need for developing new strategies to effectively and efficiently prevent and control disease spread (37–40). Some of the challenges associated with bTB diagnosis in swine include presence of fewer bacilli in bTB lesions compared to other species (41, 42), relatively high susceptibility to avian TB infection, being those lesions undistinguishable from those produced by *M. bovis* (20, 32), and the lack, despite some promising advances (43), of simple, affordable, and sensitive diagnostic tools to identify infected animals at the farm level, like the PPD test use routinely in cattle (43–45). Here, we provided evidence suggesting that the PCR may be used as an effective tool for the rapid and effective detection of the infection in swine routinely inspected at slaughterhouses in Argentina. Ultimately, results presented here will help to inform decisions intended to update control strategies in endemic settings.

The high frequency of *M. bovis* infection in bTB-like lesions estimated in this assay (74.39%), suggests that the disease continues to be prevalent in swine populations of Argentina. Furthermore, the figure is substantially higher compared to those reported elsewhere (12, 32), who detected MTC in 32.7% of TBL samples from South West Iberian Peninsula and absence of MTC positive results from samples obtained in Sao Paulo region, respectively. Still, the high frequency (31%) of bTB reported in wild boar samples from the south of Brazil (46) would suggest the need for further studies, increasing the sample size, and targeting specific risk populations. The relative high bTB prevalence in Argentine swine is likely associated with different rearing systems. Such conclusion is also supported by the observation that the risk for bTB is relatively high in pigs reared in extensive, small (<50 sow) farms that are co-located with cattle (unpublished data). In this regard, unfortunately, not much evidence has been reported regarding the interspecies dynamic of transmission of bovine tuberculosis in farms where the cohabitation with other species, like cattle, exits. A report from the Association of Veterinarians of Buenos Aires province (47) provided preliminary evidence that supports this hypothesis, describing a different frequency of slaughterhouse findings of TBL based on rearing system being more frequent in extensive farms. Biosecurity in extensive farm is usually less strict than in intensive systems, increasing the risk for bTB (48).

The Se of the PCR test estimated here (83%) is similar to that reported for cattle (10), and higher than values previously reported for pig (12). The difference may be explained, at least in part, by differences in the study design, given that previous studies have used BC as the gold standard, whereas we have used a Bayesian approach that does not make use of such a questionable assumption. Another explanation may be that we used a larger sample size (4–7 g range of homogenized tissue for initial extraction) compared to previous studies (10, 12, 49). Increasing the volume of the samples may have increased the analytical Se (and thus the diagnostic test Se) of the test, without affecting the test Sp (13). One study (12) reported a detection rate of 77.3% of culture positive samples by RT-PCR and others (31) showed a Se of 66.1%, compared to 80.1% obtained in this work using direct PCR. Only one other study (49), reported a frequency of PCR-positive results similar to ours, working with bovine samples.

The bTB control program in Argentine swine is voluntary and its implementation is based on the use of PPD test at farm level and identification of bTB-like lesions in swine without the use of a confirmatory test. Here, we estimated that ~25% of the bTB-like lesions observed at the time of slaughtering in swine are non-infected, suggesting a Sp of the inspection of ~75% (95% CI: 63–85%). Furthermore, results suggest that the PCR may be used as a rapid, effective, confirmatory test for bTB-like lesions detected in pigs at the time of slaughtering, given that the test accuracy was similar (and on average, slightly higher) to that reported for the BC. Furthermore, the significant reduction of the turnaround time between the sample submission and the result would facilitate the follow up actions on positive cases by the sanitary authority. As expected, in-series combination of the tests impaired the combined Se value (66.6%), making it not suitable as a screening protocol. Conversely, in scenarios of relatively high bTB prevalence, such as those observed in Argentina, the in-parallel use of the BC and PCR (Figure 1) showed a good performance suggesting that the combined used of those techniques would be appropriate for the confirmation of the disease in bTB-like lesions. Consequently, it is recommended that bTB-like lesions were run by PCR and the positive samples be considered bTB infected, whereas negative samples would be run by BC, and considered as infected if positive, and non-infected otherwise. Moreover, the NPV (probability that a sample negative to a screening test was non-infected) and PPV (probability that a sample positive to a screening test was infected) of the in parallel use of both tests (Figure 1) showed that this combination would be suitable for a wide range of prevalence. The combination of both tests showed much better NPV than the individual tests (% of increase, 8.5–35.3%) with prevalence values higher than 0.4; however, the combined used of the tests did not substantially impact the PPV, showing <1–8.5% reductions. Similar findings were also reported elsewhere (50–52). Results suggest that the combined use of those techniques would be appropriate for disease confirmation in bTB-like lesions and in the context of a TB control plan. The use of PCR as a routine confirmatory technique is commonly questioned taking into account only the associated direct costs (53). However, direct application of the PCR technique in tissues brings certain benefits such as the reduction of both the laboratory turnaround time and indirect costs associated with maintenance of personnel and facilities. Furthermore, bacteriological culture required significant investments in personnel training and the biosafety protocols and facilities, as it represents a much greater risk of exposure to the agent (Table 1).

In conclusion, these results suggest that bTB is still highly prevalent in swine populations of Argentina, and that the PCR may serve as an effective and rapid test for the confirmation of the agent in bTB-like lesions macroscopically detected at the time of slaughtering in the country. The results here may ultimately help to update current strategies used to prevent and control of the disease in settings in which the disease is yet-to-be eradicated.

## Author Contributions

SB, MP, and AP conceived and performed the statistical analysis. SB and MP drafted the manuscript. MJM, MM, NA, MZ, and SB participated in the generation, collection and curation of the data, and collaborated in interpretation of the results. SB and AP designed the study and coordinated the work. All authors read and approved the final manuscript.

### Conflict of Interest Statement

The authors declare that the research was conducted in the absence of any commercial or financial relationships that could be construed as a potential conflict of interest.

## References

[1] Brosch R Gordon SV Marmiesse M Brodin P Buchrieser C Eiglmeier K. A new evolutionary scenario for the *Mycobacterium tuberculosis* complex. Proc Natl Acad Sci USA. (2002) 99:3684–9. 10.1073/pnas.052548299 11891304 PMC122584

[2] Olea-Popelka F Muwonge A Perera A Dean AS Mumford E Erlacher-Vindel E. Zoonotic tuberculosis in human beings caused by *Mycobacterium bovisâ*€”a call for action. Lancet Infect Dis. (2016) 17:e21–5. 10.1016/S1473-3099(16)30139-6 27697390

[3] Katale BZ Mbugi EV Kendal S Fyumagwa RD Kibiki GS Godfrey-Faussett P Bovine tuberculosis at the human-livestock-wildlife interface: is it a public health problem in Tanzania? A review the onderstepoort. J Vet Res. (2012) 79:463 10.4102/ojvr.v79i2.463 23327384

[4] Robinson PA. A history of bovine tuberculosis eradication policy in Northern Ireland. Epidemiol Infect. (2015) 143:3182–95. 10.1017/S0950268815000291 25778830 PMC9150964

[5] McCallan L Brooks C Couzens C Young F McNair J Byrne AW. Assessment of serological tests for diagnosis of bovine tuberculosis. Vet Rec. (2017) 181:90. 10.1136/vr.104272 28386030

[6] Gormley E Corner LAL. Pathogenesis of *Mycobacterium bovis* infection: the badger model as a paradigm for understanding tuberculosis in animals. Front Vet Sci. (2017) 4:247. 10.3389/fvets.2017.00247 29379792 PMC5775213

[7] Gormley AM Anderson DP Nugent G. Cost-based optimization of the stopping threshold for local disease surveillance during progressive eradication of tuberculosis from New Zealand wildlife. Transbound Emerg Dis. (2018) 65:186–96. 10.1111/tbed.12647 28391623

[8] Lahuerta-Marin A Milne MG McNair J Skuce RA McBride SH Menzies FD. Bayesian latent class estimation of sensitivity and specificity parameters of diagnostic tests for bovine tuberculosis in chronically infected herds in Northern Ireland. Vet J. (2018) 238:15–21. 10.1016/j.tvjl.2018.04.019 30103911

[9] Schiller I Oesch B Vordermeier HM Palmer MV Harris BN Orloski KA. Bovine tuberculosis: a review of current and emerging diagnostic techniques in view of their relevance for disease control and eradication. Transbound Emerg Dis. (2010) 57:205–20. 10.1111/j.1865-1682.2010.01148.x 20561288

[10] Courcoul A Moyen JL Brugère L Faye S Hénault S Gares H. Estimation of sensitivity and specificity of bacteriology, histopathology and PCR for the confirmatory diagnosis of bovine tuberculosis using latent class analysis. PLoS ONE. (2014) 9:e90334. 10.1371/journal.pone.0090334 24625670 PMC3953111

[11] de la Rua-Domenech R Human *Mycobacterium bovis* infection in the United Kingdom: incidence, risks, control measures and review of the zoonotic aspects of bovine tuberculosis. Tuberculosis. (2006) 86:77–109. 10.1016/j.tube.2005.05.002 16257579

[12] Cardoso-Toset F Luque I Amarilla SP Gómez-Gascón L Fernández L Huerta B. Evaluation of rapid methods for diagnosis of tuberculosis in slaughtered free-range pigs. Vet J. (2015) 204:232–4. 10.1016/j.tvjl.2015.01.022 25920761

[13] Fell S Bröckl S Büttner M Rettinger A Zimmermann P Straubinger RK. Two alternative DNA extraction methods to improve the detection of Mycobacterium-tuberculosis-complex members in cattle and red deer tissue samples. BMC Microbiol. (2016) 16:213. 10.1186/s12866-016-0816-2 27629399 PMC5024493

[14] Taylor GM Worth DR Palmer S Jahans K Hewinson RG. Rapid detection of *Mycobacterium bovis* DNA in cattle lymph nodes with visible lesions using PCR. BMC Vet Res. (2007) 3:12. 10.1186/1746-6148-3-12 17567891 PMC1904440

[15] Parra A García N García A Lacombe A Moreno F Freire F. Development of a molecular diagnostic test applied to experimental abattoir surveillance on bovine tuberculosis. Vet Microbiol. (2008) 127:315–24. 10.1016/j.vetmic.2007.09.001 17954014

[16] Araújo CP Osório ALAR Jorge KSG Ramos CAN Souza Filho AF Vidal CES. Direct detection of *Mycobacterium tuberculosis* complex in bovine and bubaline tissues through nested-PCR. Braz J Microbiol. (2014) 45:633–40. 10.1590/S1517-83822014000200035 25242951 PMC4166292

[17] Torres P Bovine Tuberculosis in Argentina, Current Situation. Buenos Aires (2016). Retrieved from: http://www.senasa.gob.ar/sites/default/files/ARBOL_SENASA/ANIMAL/BOVINOS_BUBALINOS/PROD_PRIMARIA/SANIDAD/ENF_Y_ESTRAT/TUBERCULOSIS/situacion_tuberculosis_bovina_rep._argentina_2015.pdf

[18] Barandiaran S Martínez Vivot M Moras EV Cataldi AA Zumárraga MJ. *Mycobacterium bovis* in Swine: spoligotyping of isolates from Argentina. Vet Med Int. (2011) 2011:979647. 10.4061/2011/979647 21547236 PMC3087618

[19] Barandiaran S Martínez Vivot M Pérez AM Cataldi AA Zumárraga MJ. Bovine tuberculosis in domestic pigs: genotyping and distribution of isolates in Argentina. Res Vet Sci. (2015) 103:44–50. 10.1016/j.rvsc.2015.09.013 26679794

[20] Barandiaran S Perez AM Giofre AK Martinez Vivot M Cataldi AA Zumarraga MJ. Tuberculosis in swine co-infected with *Mycobacterium avium* subsp. hominissuis and Mycobacterium bovis in a cluster from Argentina. Epidemiol Infect. (2015)143:966–74. 10.1017/S095026881400332X 25496827 PMC9507158

[21] de la Rua-Domenech R Goodchild AT Vordermeier HM Hewinson RG Christiansen KH Clifton-Hadley RS. Ante mortem diagnosis of tuberculosis in cattle: a review of the tuberculin tests, γ-interferon assay and other ancillary diagnostic techniques. Res Vet Sci. (2006) 81:190–210. 10.1016/j.rvsc.2005.11.005 16513150

[22] de Lisle GW Bengis RG Schmitt SM O'Brien DJ. Tuberculosis in free-ranging wildlife: detection, diagnosis and management. Revue Sci Tech. (2002) 21:317–34. 10.20506/rst.21.2.1339 11974618

[23] Buddle BM de Lisle GW Griffin JFT Hutchings SA. Epidemiology, diagnostics, and management of tuberculosis in domestic cattle and deer in New Zealand in the face of a wildlife reservoir. N Z Vet J. (2015) 63 (Suppl 1):19–27. 10.1080/00480169.2014.929518 24992203 PMC4566881

[24] Branscum AJ Gardner IA Johnson WO. Estimation of diagnostic-test sensitivity and specificity through Bayesian modeling. Prevent Vet Med. (2005) 68:145–63. 10.1016/j.prevetmed.2004.12.005 15820113

[25] Al-Mouqatea S Alkhamis M Akbar B Ali A Al-Aqeel H Bin-Heji A. Bayesian estimation of ELISA and gamma interferon test accuracy for the detection of bovine tuberculosis in caudal fold test-negative dairy cattle in Kuwait. J Vet Diagnost Invest. (2018) 30:468–70. 10.1177/1040638718759574 29431048 PMC6505825

[26] Latini O Amadio G Di Lonardo M Barrera L Kantor I Metodos Bacteriologicos Empleados En El Diagnostico de la Tuberculosis en la Argentina: Evaluacion de la Calidad. Boletín de la Oficina Sanitaria Panamericana (OSP);104. (1988). Retrivered from: http://iris.paho.org/xmlui/handle/123456789/15779

[27] Hermans PW Schuitema AR Van Soolingen D Verstynen CP Bik EM Thole JE. Specific detection of *Mycobacterium tuberculosis* complex strains by polymerase chain reaction. J Clin Microbiol. (1990) 28:1204–13. 2116445 10.1128/jcm.28.6.1204-1213.1990 PMC267906

[28] Zumárraga MJ Meikle V Bernardelli A Abdala A Tarabla H Romano MI. Use of touch-down polymerase chain reaction to enhance the sensitivity of *Mycobacterium bovis* detection. J Vet Diagnost Invest. (2005) 17:232–8. 10.1177/104063870501700303 15945378

[29] Kamerbeek J Schouls L Kolk A van Agterveld M van Soolingen D Kuijper S. Simultaneous detection and strain differentiation of Mycobacterium tuberculosis for diagnosis and epidemiology. J Clin Microbiol. (1997) 35:907–14. 9157152 10.1128/jcm.35.4.907-914.1997 PMC229700

[30] Guerrero C Bernasconi C Burki D Bodmer T Telenti A. A novel insertion element from *Mycobacterium avium*, IS1245, is a specific target for analysis of strain relatedness. J Clin Microbiol. (1995) 33:304–7. 7714183 10.1128/jcm.33.2.304-307.1995 PMC227937

[31] Santos N Geraldes M Afonso A Almeida V Correia-Neves M. Diagnosis of tuberculosis in the wild boar (sus scrofa): a comparison of methods applicable to hunter-harvested animals. PLoS ONE. (2010) 5:12663. 10.1371/journal.pone.0012663 20844754 PMC2937024

[32] Lara GHB Ribeiro MG Leite CQF Paes AC Guazzelli A Silva AV. Occurrence of Mycobacterium spp. and other pathogens in lymph nodes of slaughtered swine and wild boars (Sus scrofa). Res Vet Sci. (2011) 90:185–8. 10.1016/j.rvsc.2010.06.009 20621319

[33] Dendukuri N Joseph L. Bayesian approaches to modeling the conditional dependence between multiple diagnostic tests. Biometrics. (2001) 57:158–67. 10.1111/j.0006-341X.2001.00158.x 11252592

[34] de Blas I Ruiz-Zarzuela I Vallejo A WinEpi: Working In Epidemiology. An Online Epidemiological Tool. In: International Symposium on Veterinary Epidemiology and Economics. Cairns, QLD: 11th ISVEE (2006).

[35] Kostoulas P Nielsen SS Branscum AJ Johnson WO Dendukuri N Dhand NK. STARD-BLCM: standards for the reporting of diagnostic accuracy studies that use bayesian latent class models. Prev Vet Med. (2017) 138:37–47. 10.1016/j.prevetmed.2017.01.006 28237234

[36] Picasso C Alvarez J VanderWaal KL Fernandez F Gil A Wells SJ. Epidemiological investigation of bovine tuberculosis outbreaks in Uruguay (2011–2013). Prev Vet Med. (2017) 138:156–61. 10.1016/j.prevetmed.2017.01.010 28237231

[37] WHO OIE FAO Roadmap for Zoonotic Tuberculosis. (p. 21). WHO Press (2017). Retrieved from: http://www.who.int/

[38] Abalos P Retamal P. Tuberculosis: a re-emerging zoonosis? Rev Sci Techn de l'OIE. (2004) 23:583–94. 10.20506/rst.23.2.1502 15702721

[39] Zumárraga MJ Arriaga C Barandiaran S Cobos-Marín L de Waard J Estrada-Garcia I. Understanding the relationship between *Mycobacterium bovis* spoligotypes from cattle in Latin American Countries. Res Vet Sci. (2013) 94:9–21. 10.1016/j.rvsc.2012.07.012 22884173

[40] Silva MR da Rocha AS Araújo FR Fonseca-Júnior AA Alencar AP de Suffys PN. Risk factors for human *Mycobacterium bovis* infections in an urban area of Brazil. Mem Do Instit Oswaldo Cruz. (2018) 113:e170445. 10.1590/0074-02760170445 29898014 PMC5989489

[41] Nugent G. Maintenance, spillover and spillback transmission of bovine tuberculosis in multi-host wildlife complexes: a New Zealand case study. Vet Microbiol. (2011) 151:34–42. 10.1016/j.vetmic.2011.02.023 21458931

[42] García-Jiménez WL Salguero FJ Fernández-Llario P Martínez R Risco D Gough J. Immunopathology of granulomas produced by *Mycobacterium bovis* in naturally infected wild boar. Vet Immunol Immunopathol. (2013) 156:54–63. 10.1016/j.vetimm.2013.09.008 24144683

[43] Thomas J Infantes-Lorenzo JA Moreno I Cano-Terriza D de Juan L García-Bocanegra I. Validation of a new serological assay for the identification of *Mycobacterium tuberculosis* complex-specific antibodies in pigs and wild boar. Prevent Vet Med. (2019) 162:11–7. 10.1016/j.prevetmed.2018.11.004 30621888

[44] Magnano GG Schneider MO Urbani CE Ambrogi A Zapata L Jorge MC Comparación de Técnicas Diagnósticas de Tuberculosis Porcina en dos Establecimientos De Cría Confinada en Argentina. InVet, 25–31 (2010). Retrieved from: http://www.scielo.org.ar/scielo.php?script=sci_arttextandpid=S1668-34982010000100004andlang=pt

[45] de la Cruz ML Branscum AJ Nacar J Pages E Pozo P Perez A. Evaluation of the performance of the IDvet IFN-Gamma test for diagnosis of bovine tuberculosis in Spain. Front Vet Sci. (2018) 5:229. 10.3389/fvets.2018.00229 30320129 PMC6171474

[46] Maciel ALG Loiko MR Bueno TS Moreira JG Coppola M Dalla Costa ER. Tuberculosis in Southern Brazilian wild boars (*Sus scrofa*): first epidemiological findings. Transbound Emerg Dis. (2018) 65:518–26. 10.1111/tbed.12734 29076653

[47] Dumrauf D Passucci G Rodriguez G Diaz M Hallazgos patologicos en la faena de cerdos adultos. Revista del Colegio de Veterinarios de la Provincia de Buenos Aires. (2015).

[48] Dibarbora M Cappuccio JA Aznar MN Bessone FA Piscitelli H Pereda AJ Detección serológica de Brucella suis, virus de influenza y virus de la enfermedad de Aujeszky en criaderos porcinos familiares de menos de 100 madres en Argentina. Rev Argent Microbiol. (2017) 49:158–65. 10.1016/j.ram.2016.09.010 28325625

[49] Stewart LD McNair J McCallan L Gordon A Grant IR. Improved detection of *Mycobacterium bovis* Infection in Bovine lymph node tissue using immunomagnetic separation (IMS)-based methods. PLoS ONE. (2013) 8:e58374. 10.1371/journal.pone.0058374 23469275 PMC3587598

[50] Toft N Åkerstedt J Tharaldsen J Hopp P. Evaluation of three serological tests for diagnosis of Maedi-Visna virus infection using latent class analysis. Vet Microbiol. (2007) 120:77–86. 10.1016/j.vetmic.2006.10.025 17118583

[51] de Arruda MM Figueiredo FB Marcelino AP Barbosa JR Werneck GL Noronha EF. Sensitivity and specificity of parallel or serial serological testing for detection of canine Leishmania infection. Mem Do Inst Oswaldo Cruz. (2016) 111:168–73. 10.1590/0074-02760150364 26910354 PMC4804499

[52] Franco F Di Napoli A Evaluation of diagnostic tests in parallel and in series. G Di Tecniche Nefrologiche e Dialitiche. (2016) 28:212–5. 10.5301/GTND.2016.15992

[53] Taylor MJ Hughes MS Skuce RA Neill SD. Detection of *Mycobacterium bovis* in bovine clinical specimens using real-time fluorescence and fluorescence resonance energy transfer probe rapid-Cycle PCR. J Clin Microbiol. (2001) 39:1272–8. 10.1128/JCM.39.4.1272-1278.2001 11283040 PMC87923

