# Supplementary material for: Bayesian Assessment of the Accuracy of a PCR-Based Rapid Diagnostic Test for Bovine Tuberculosis in Swine
Source: Front Vet Sci. 2019 Jun 26;6:204. doi: 10.3389/fvets.2019.00204 (PMC6608602; doi:10.3389/fvets.2019.00204)
Supplement: Supplementary file 1 [file Table_1.DOCX]

**Supplementary table 1.** Results from the sensitivity analyses using uniform distributed priors for both sensitivities (Secul, Sepcr), both specificities (Spcul, Sppcr), and Prevalence (pi). Reference values indicate posterior median results, and posterior probability intervals (95% PPI). Var: percentage of variation between both estimations.

|  | Posterior distributions | | | | | | | | | | |
| --- | --- | --- | --- | --- | --- | --- | --- | --- | --- | --- | --- |
|  | Model with informative priors | Secul~unif (0,1) | Var  % | Sepcr~unif (0,1) | Var  % | Spcul~unif (0,1) | Var  % | Sppcr~unif (0,1) | Var  % | pi~unif  (0,1) | Var  % |
| Secul | **79.9%**  **(71.69-88.7%)** | 80.6%  (71.33-96.1%) | 0.87 | 79.83%  (71.69-88.6%) | -0.09 | 76.42%  (66.21-85.5%) | -4.35 | 85.02%  (74.2-94.1%) | 6.8 | 77.4%  (66.68-86.1%) | -3.13 |
| Sepcr | **82.9%**  **(74.35-92.3%)** | 82.68%  (74.3-92.3%) | -0.3 | 83.65%  (73.98-98.1%) | 0.9 | 87.82%  (77.1-96.7%) | 5.93 | 78.51%  (67.1-89.6%) | -5.3 | 79.68%  (68.65-88.8%) | -3.88 |
| Spcul | **88.5%**  **(67.2-99.5%)** | 91.1%  (67.4-99.56%) | 2.9 | 88.8%  (63.1-99.4%) | 0.3 | 64.95%  (42.1-93.91%) | **-26.6** | 90.86%  (66-99.5%) | 2.66 | 93.88%  (73.2-99.68%) | 6.06 |
| Sppcr | **89.05%**  **(69.8-99.1%)** | 89.46%  (74.5-99.34%) | 0.5 | 91.16%  (70.1-99.17%) | 2.36 | 90.52%  (69.6-99.06%) | 1.65 | 63.07%  (37.9-94.5%) | **-29.1** | 92.89%  (74.5-99.34%) | 3.48 |
| pi | **74.39%**  **(63.3-83.5%)** | 74.08%  (60.02-83.5%) | -0.41 | 74%  (60.3-83.6%) | -0.52 | 69.87%  (57.3-81.12%) | -6.07 | 69.77%  (55.8-81%) | -6.2 | 80.55%  (69.4-95.35%) | 8.28 |
