# Supplementary material for: Bayesian Assessment of the Accuracy of a PCR-Based Rapid Diagnostic Test for Bovine Tuberculosis in Swine
Source: Front Vet Sci. 2019 Jun 26;6:204. doi: 10.3389/fvets.2019.00204 (PMC6608602; doi:10.3389/fvets.2019.00204)
Supplement: Supplementary file 2 [file Table_2.DOCX]

model{

x[1:4] ~ dmulti(p[1:4], n)

p[1] <- pi*(Secul*Sepcr+covDp) + (1-pi)*((1-Spcul)*(1-Sppcr)+covDn)

p[2] <- pi*(Secul*(1-Sepcr)-covDp) + (1-pi)*((1-Spcul)*Sppcr-covDn)

p[3] <- pi*((1-Secul)*Sepcr-covDp) + (1-pi)*(Spcul*(1-Sppcr)-covDn)

p[4] <- pi*((1-Secul)*(1-Sepcr)+covDp) + (1-pi)*(Spcul*Sppcr+covDn)

ls <- (Secul-1)*(1-Sepcr)

us <- min(Secul,Sepcr) - Secul*Sepcr

lc <- (Spcul-1)*(1-Sppcr)

uc <- min(Spcul,Sppcr) - Spcul*Sppcr

pi ~ dbeta(5.025, 7.0375) ### Mode=0.32, 95% sure > 0.20

Secul ~ dbeta(16.1034, 5.0197) ### Mode=0.79, 99% sure > 0.6

Spcul ~ dbeta(14.5219, 1.1365) ### Mode=0.99, 99% sure > 0.8

Sepcr ~ dbeta(13.776, 3.7573) ### Mode=0.81, 99% sure > 0.5

Sppcr ~ dbeta(17.297, 1.5041) ### Mode=0.98, 99% sure > 0.8

covDn ~ dunif(lc, uc)

covDp ~ dunif(ls, us)

rhoD <- covDp / sqrt(Secul*(1-Secul)*Sepcr*(1-Sepcr))

rhoDc <- covDn / sqrt(Spcul*(1-Spcul)*Sppcr*(1-Sppcr))

}

list(n=266, x=c(137,34,39,56))

list(pi=0., Secul=0.79, Spcul=0.97, Sepcr=0.81, Sppcr=0.99)
