# Supplementary material for: Bayesian Assessment of the Accuracy of a PCR-Based Rapid Diagnostic Test for Bovine Tuberculosis in Swine
Source: Front Vet Sci. 2019 Jun 26;6:204. doi: 10.3389/fvets.2019.00204 (PMC6608602; doi:10.3389/fvets.2019.00204)

Supplementary Figure 1. Visual inspection of the Markov chains (Secul= Sensitivity of the bacteriological culture; Spcul= Specificity of the bacteriological culture; Sepcr= Sensitivity of the PCR; Sppcr= Specificity of the PCR; pi= prevalence of *M.bovis* on bTB-like lesions).

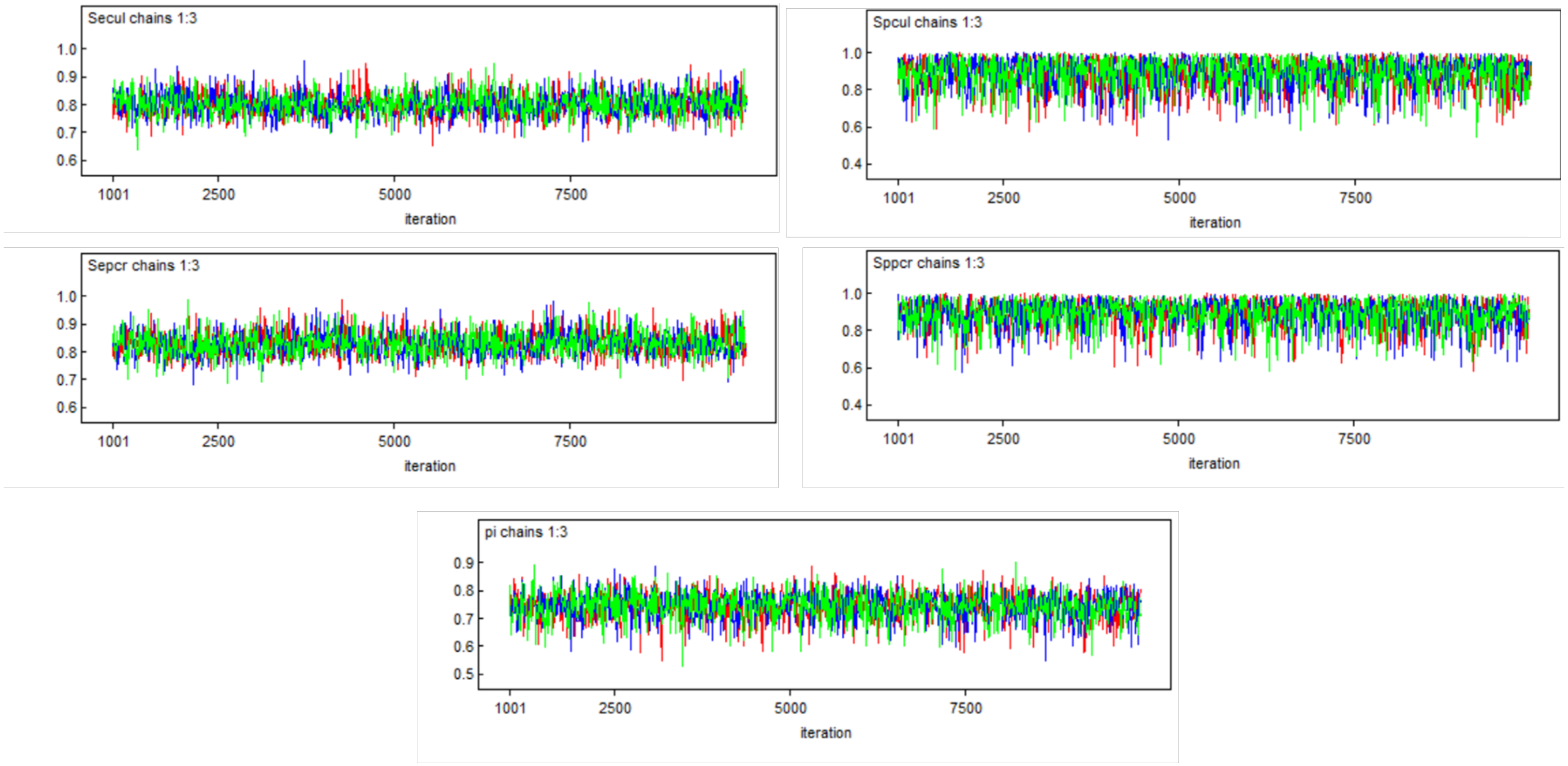

Supplement: Supplementary file 3 [file Data_Sheet_1.PDF]
